# Author Correction: Targeting adipocyte ESRRA promotes osteogenesis and vascular formation in adipocyte-rich bone marrow

**DOI:** 10.1038/s41467-025-56599-y

**Published:** 2025-02-03

**Authors:** Tongling Huang, Zhaocheng Lu, Zihui Wang, Lixin Cheng, Lu Gao, Jun Gao, Ning Zhang, Chang-An Geng, Xiaoli Zhao, Huaiyu Wang, Chi-Wai Wong, Kelvin W. K. Yeung, Haobo Pan, William Weijia Lu, Min Guan

**Affiliations:** 1 Research Center for Human Tissues and Organs Degeneration, Institute of Biomedicine and Biotechnology, Shenzhen Institute of Advanced Technology, Chinese Academy of Sciences, Shenzhen, China; 2 University of Chinese Academy of Sciences, Beijing, China; 3 Guangdong Provincial Clinical Research Center for Geriatrics, Shenzhen Clinical Research Center for Geriatrics, Shenzhen People’s Hospital, Shenzhen, China; 4 Neuroscience Center, Shantou University Medical College, Shantou, China; 5 State Key Laboratory of Phytochemistry and Plant Resources inWest China, Kunming Institute of Botany, Chinese Academy of Sciences, Kunming, China; 6 Guangzhou Huazhen Biosciences, Guangzhou, China; 7 Department of Orthopaedics and Traumatology, Li Ka Shing Faculty of Medicine, The University of Hong Kong, Hong Kong, China; 8 Faculty of Pharmaceutical Sciences, Shenzhen Institute of Advanced Technology, Chinese Academy of Sciences, Shenzhen, China

Correction to: *Nature Communications* 10.1038/s41467-024-48255-8, published online 04 May 2024

The original version of this Article contained errors in Fig. 7 panels e and i, which occurred during final preparation of the figures and don’t affect the conclusions. The image shown in panel 7e “+rLeptin” was mistakenly duplicated in panel 7i “+SPP1 Nab&rLeptin”.

The correct version of Fig. 7e and i is:
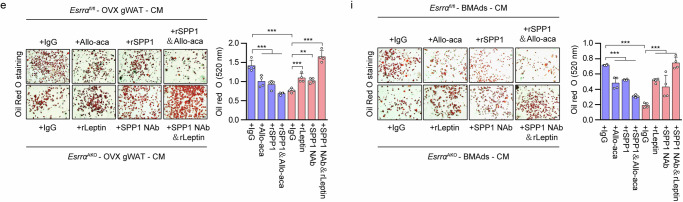


Which replaces the incorrect version of Fig. 7 e and i:
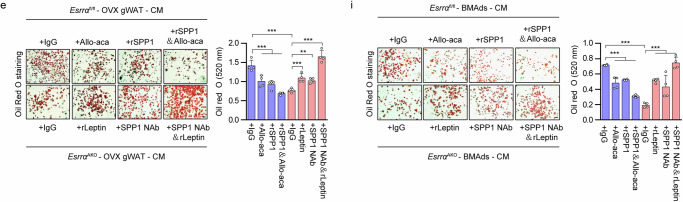


This has been corrected in both the PDF and HTML versions of the Article.

