# Supplementary material for: Targeting adipocyte ESRRA promotes osteogenesis and vascular formation in adipocyte-rich bone marrow
Source: Nat Commun. 2024 May 4;15:3769. doi: 10.1038/s41467-024-48255-8 (PMC11069533; doi:10.1038/s41467-024-48255-8)
Supplement: Supplementary file 1 — Supplementary Information [file 41467_2024_48255_MOESM1_ESM.pdf]

Title: Targeting adipocyte ESRRA promotes osteogenesis and vascular formation in adipocyte-rich bone marrow

Supplementary information contents:

1. Supplementary Figures: 1-8
2. Supplementary Tables: 1-3

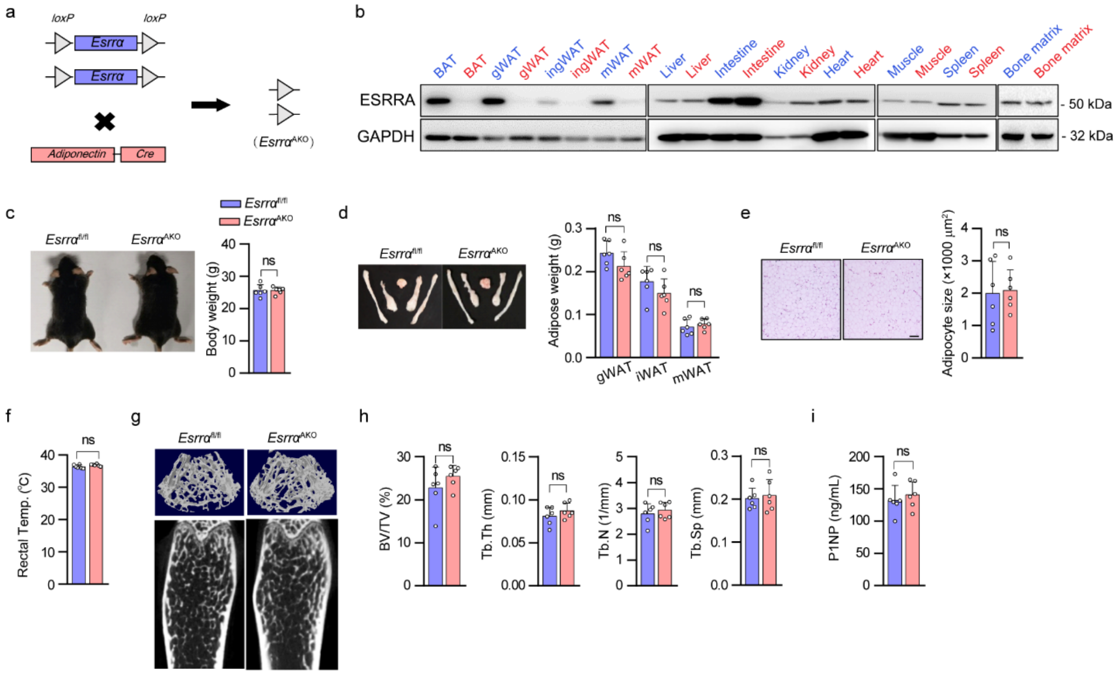

Supplementary Figure 1. *Esrra*^{AKO} male mice exhibit normal WAT and bone phenotype at the age of 10-week-old. a Schematic diagram showing the breeding strategy to generate adipocyte-specific knockout *Esrra* mice. *Esrra*$^{\text{flox/flox}}$ mice were continuously mated with AdipoqCre mice to obtain *Esrra*$^{\text{flox/flox}}$; AdipoCre mice (*Esrra*^{AKO}) and littermate control *Esrra*$^{\text{flox/flox}}$ mice (*Esrra*$^{\text{fl/fl}}$). Phenotypic analysis was conducted on 10-week-old mice. **b** The protein expression levels of ESRRA were evaluated in adipose tissues and non-adipose tissues, comparing *Esrra*$^{\text{fl/fl}}$ mice (blue font) with *Esrra*^{AKO} mice (red font). **c** Representative images and quantitative analysis of body weights in *Esrra*$^{\text{fl/fl}}$ and *Esrra*^{AKO} male mice. **d** Representative images of white adipose tissue depots. The weights of gonadal WAT (gWAT), inguinal WAT (iWAT), and mesentery WAT (mWAT) are shown as graphs. **e** H&E staining of gWAT sections and quantitative analysis of adipocyte size (scale bar: 50 μm). **f** Rectal temperatures of the mice at room temperatures (24 ± 2°C). **g** Representative single micro-CT sagittal section and 3-dimensional reconstitution of distal femurs. **h** Quantitative micro-CT analysis of BV/TV, Tb.Th, Tb.N and Tb.Sp. **i** Plasma P1NP levels. Data are shown as mean ± SD (n = 6 mice per group). Statistical analysis is performed using unpaired two-tailed Student's t-test. Source data are provided as a Source Data file.

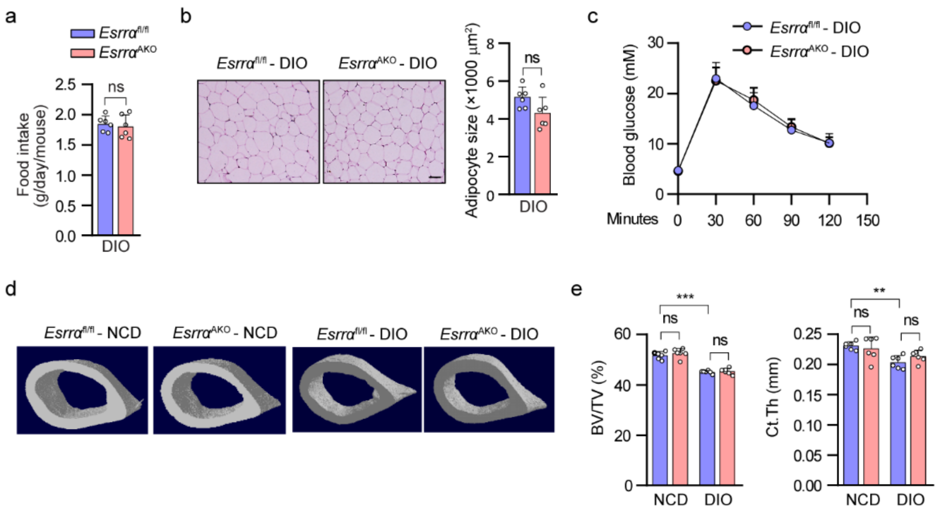

Supplementary Figure 2. Adipocyte ESRRA ablation results in no alterations in WAT and cortical bone in 25-week-old NCD and DIO mice. a Daily food intake in HFD-fed *Esrra*$^{fl/fl}$ and *Esrra*^{AKO} mice. **b** H&E-stained gWAT sections (scale bar: 50 μm). The areas of adipocytes size are presented as graphs. **c** Oral glucose tolerance test (OGTT) was analyzed in *Esrra*$^{fl/fl}$ and *Esrra*^{AKO} mice fed a HFD for 12 weeks. **d** Representative μCT images of cortical bone in femurs from *Esrra*$^{fl/fl}$ and *Esrra*^{AKO} mice fed a NCD and HFD. **e** Bone histomorphometric analysis of BV/TV and Ct.Th of cortical bone in femoral midshaft. Data are shown as mean ± SD (n = 6 mice per group). **, $P < 0.01$ and ***, $P < 0.001$. Statistical analysis is performed using unpaired two-tailed Student's t-test (**a**, **b** and **c**) and two-way ANOVA with Fisher's LSD post hoc analysis (**e**). Source data are provided as a Source Data file.

.

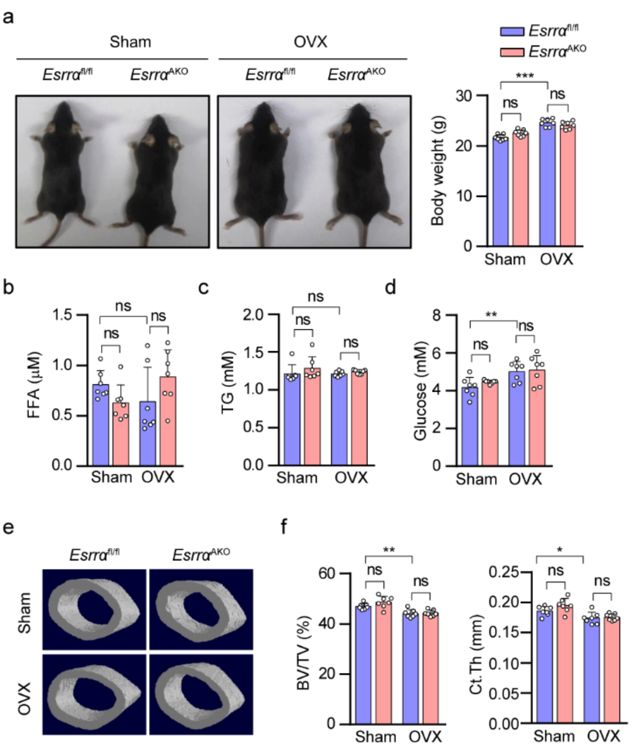

Supplementary Figure 3. *Esrra*^{AKO} female mice display no significant changes in blood biochemistry and cortical bone compared to *Esrra*$^{fl/fl}$ mice following OVX. **a** Representative photographs and body weights analysis of *Esrra*$^{fl/fl}$ and *Esrra*^{AKO} female mice underwent either sham or OVX operation for 8 weeks. **b-c** Plasma FFA (**b**) and TG (**c**) levels. **d** Blood glucose levels. **e-f** Representative μCT images (**e**) and bone histomorphometric analysis of BV/TV and Ct.Th (**f**) of cortical bone in femoral midshaft. Data are shown as mean ± SD (n = 7 mice per group). *, $P < 0.05$, **, $P < 0.01$ and ***, $P < 0.001$. Statistical analysis is performed using two-way ANOVA with Fisher's LSD post hoc analysis. Source data are provided as a Source Data file.

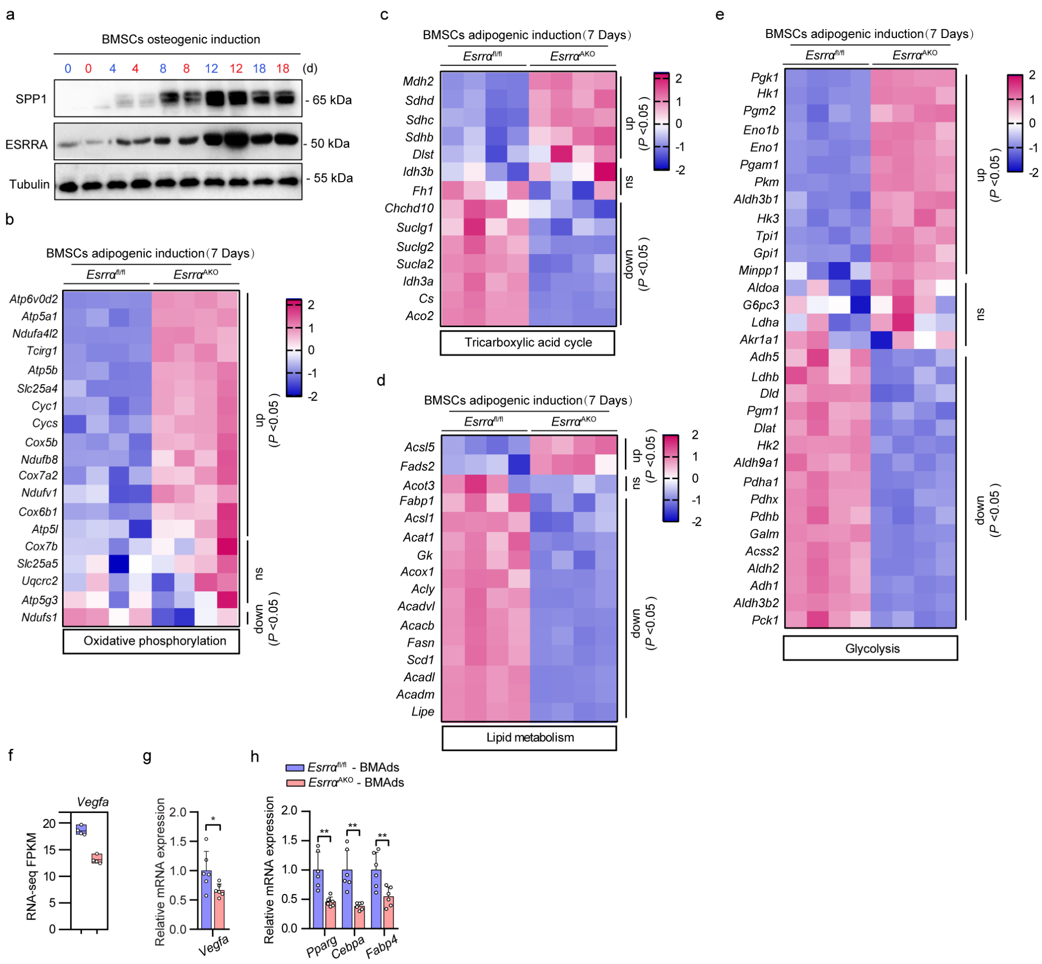

Supplementary Figure 4. The relative expression of genes in comparisons in adipocyte-lineage cells derived from BMSCs between experimental groups. a Protein expression levels of ESRRA and SPP1 were evaluated in BMSCs from *Esrra*$^{fl/fl}$ mice (blue font) and *Esrra*^{AKO} mice (red font) at the indicated days upon osteogenic induction. **b-e** Heat map depicting relative expression of oxidative phosphorylation genes (**b**), tricarboxylic acid cycle genes (**c**), lipid metabolism genes (**d**) and glycolysis genes (**e**) across two experimental group comparisons in adipocyte-lineage cells from *Esrra*^{AKO} and *Esrra*$^{fl/fl}$ mice. Differentially expressed genes were identified using DESeq2 analysis (n = 4 biologically independent samples, $P < 0.05$). **f** Boxplot showing the transcript expression value (FPKM) of *Vegfa* based on RNA-seq data (n = 4 biologically independent samples). Data are represented as box and whiskers with bars representing maximum and minimum values and with median highlighted as a line. **g** Validation of *Vegfa* mRNA expression is performed by qRT-PCR in BMAds that were

fully differentiated for 14 days. mRNA expression levels of adipogenic markers *Pparg, Cebpa* and *Fabp4* were measured in BMAds from *Esrra*$^{fl/fl}$ and *Esrra*^{AKO} mice. n = 6 mice per group. Data are shown as mean ± SD. *, $P < 0.05$, **, $P < 0.01$ and ***, $P < 0.001$. Statistical analysis is performed using unpaired two-tailed Student's t-test. Source data are provided as a Source Data file.

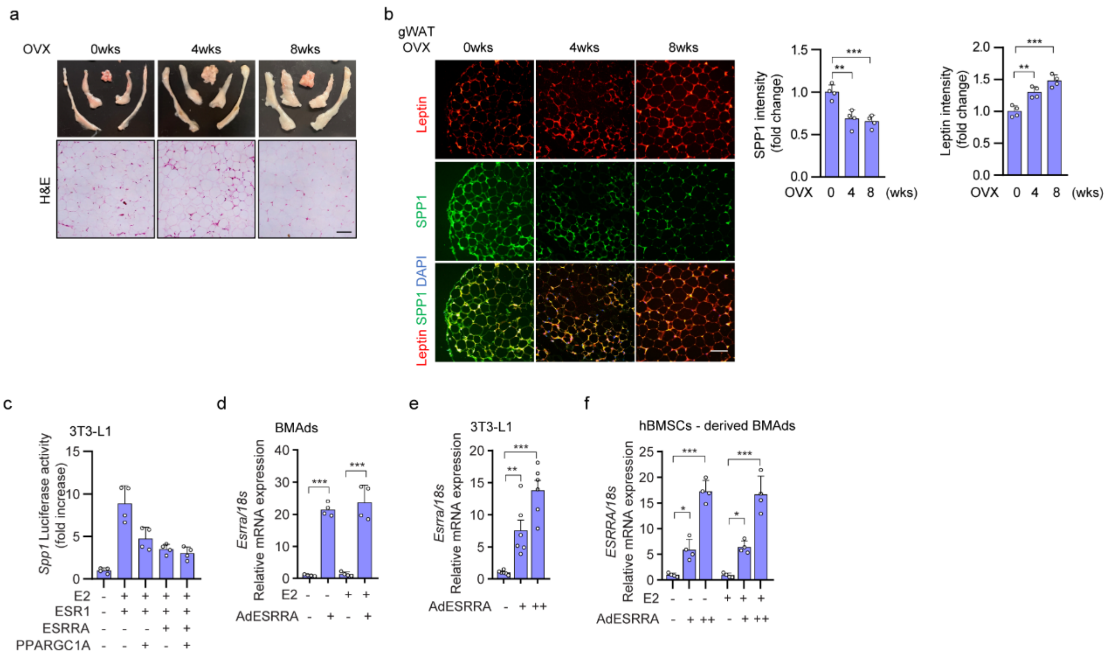

Supplementary Figure 5. ESRRA represses the transcriptional activity of *Spp1* in adipocytes. a Representative images of white adipose depots were obtained from wild-type mice at 0 week, 4 weeks, and 8 weeks after OVX surgery. **b** Immunofluorescence analysis of SPP1 and leptin in gWAT of wild-type female mice following OVX. Scale bar: 50 µm. n = 4 mice per group. **c** *Spp1* luciferase assay in 3T3-L1 cells cotransfected with ESRRA and/or PPARGC1A expression vectors, along with the ESR1 expression vector, and treated with E2 or DMSO for 48 hours during adipogenic induction. **d-f** mRNA expression levels of *Esrra* were measured in murine BMAds (**d**), matured 3T3-L1 adipocytes (**e**), and human BMSCs-derived BMAds (**f**) infected with adenovirus expressing ESRRA or control GFP in the presence of the indicated treatments of E2 (10 nM) for 2 days. n = 4 independent experiments (**d,f**) and n = 6 independent experiments (**e**). Data are shown as mean ± SD. *, $P < 0.05$ and **, $P < 0.01$. Statistical analysis is performed using one-way ANOVA followed by Bonferroni's post hoc tests. Source data are provided as a Source Data file.

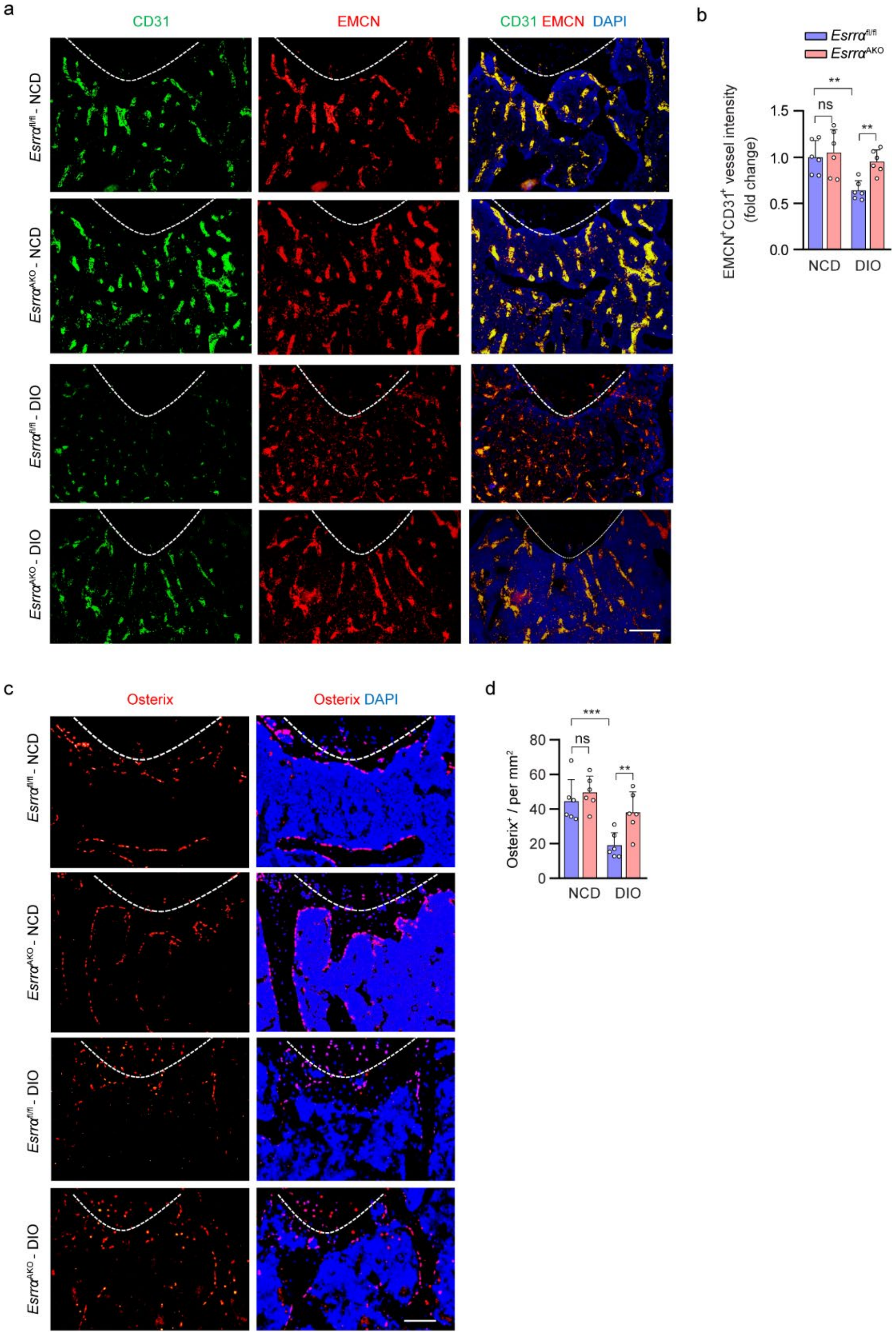

Supplementary Figure 6. Immunofluorescence staining for type H vessel and osteoprogenitors in distal femurs of *Esrra*^{AKO} NCD and DIO mice compared to corresponding control mice. a-b Representative images and quantitation analysis of metaphyseal type H vessels immunostained for EMCN (red) and CD31 (green) in distal femurs of *Esrra*$^{\mathrm{fl/fl}}$ and *Esrra*^{AKO} mice fed a NCD or HFD. DAPI (blue) is used for counterstaining of nuclei. Scale bar: 100 μm. **c-d** Immunostaining and quantitation

analysis of Osterix (red) with DAPI (blue) in the metaphysis of distal femurs. Scale bar: 50 μm. Data are shown as mean ± SD (n = 6 mice per group). **, $P < 0.01$ and ***, $P < 0.001$. Statistical analysis is performed using two-way ANOVA with Fisher's LSD post hoc analysis (**b** and **d**). Source data are provided as a Source Data file.

.

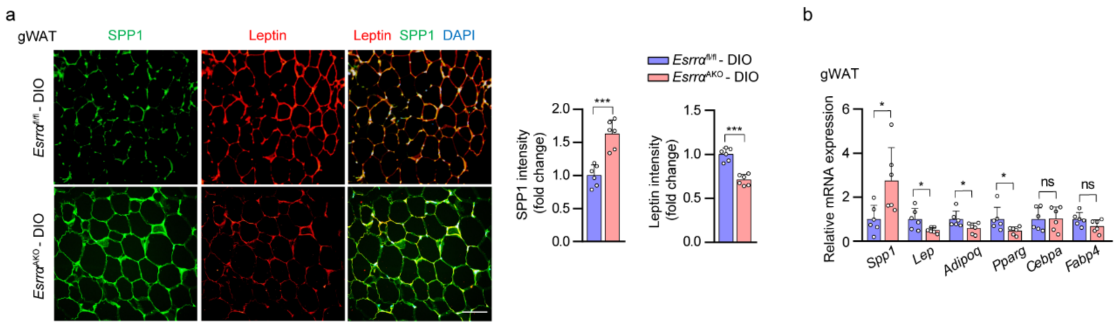

Supplementary Figure 7. Immunofluorescence and mRNA levels analysis in gWAT of *Esrra*^{AKO} DIO mice and corresponding control mice. a Immunofluorescence co-staining of SPP1 and leptin with quantitative analysis of fluorescence intensity in gWAT of *Esrra*$^{fl/fl}$ - DIO and *Esrra*^{AKO} - DIO mice (scale bar: 100 μm). **b** mRNA expression of *Spp1, Lep, Pparg, Cebpa* and *Fabp4* were examined in gWAT. Data are shown as mean ± SD (n = 6 mice per group). *, $P < 0.05$ and ***, $P < 0.001$.Statistical analysis is performed using unpaired two-tailed Student's t-test. Source data are provided as a Source Data file.

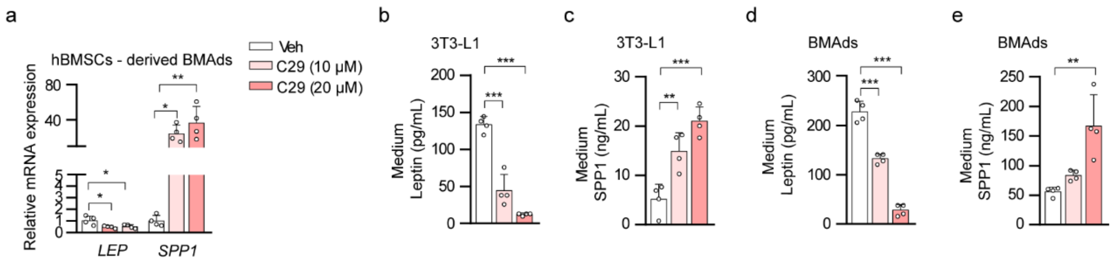

Supplementary Figure 8. mRNA expression and secretion levels of SPP1 and leptin in adipocytes treated with C29. **a** mRNA levels of *LEP* and *SPP1* were measured in human BMSCs-derived BMAds treated with C29 or DMSO for 2 days. n = 4 biologically independent experiments. **b-c** The concentrations of soluble SPP1 (**b**) and leptin (**c**) in the culture medium of mature 3T3-L1 adipocytes were measured by ELISA (n = 4 biologically independent samples). **d-e** The concentrations of soluble SPP1 (**d**) and leptin (**e**) in BMAds-CM (n = 4 biologically independent samples). Data are shown as mean ± SD. *, $P < 0.05$, **, $P < 0.01$ and ***, $P < 0.001$. Statistical analysis is performed using one-way ANOVA followed by Bonferroni's post hoc tests. Source data are provided as a Source Data file.

Supplementary Table 1. Primer sequences of promoter vectors

Primer name	Sequence 5’- 3’
Spp1-WT-luc	F: CGGGGTACCGGGGTCATATGGTTCAGCTC<br>R: CCCAAGCTTAGACTGCAAACCCAAGCAAG
Spp1-ΔR2-luc	F: CGGGGTACCGGGTCTCACTTTCTGTTGCC<br>R: CCCAAGCTTAGACTGCAAACCCAAGCAAG
Leptin-WT-luc	F: CTAGCTAGCTGGGCATGATGCGACCATTT<br>R: CCCAAGCTTAGCTGCTGGAGCAGGGA
Leptin-S123-luc	F: CTAGCTAGCCTTCGGGTACCAAAGGAAGACA<br>R: CCCAAGCTTAGCTGCTGGAGCAGGGAT
Leptin-S12-luc	F: CTAGCTAGCCCTCTGAGCAGCCAGGTTAGG<br>R: CCCAAGCTTAGCTGCTGGAGCAGGGAT
Leptin-S1-luc	F: CTAGCTAGCGCAAAGAGCTGTCGGAAAAA<br>R: CCCAAGCTTAGCTGCTGGAGCAGGGAT
Leptin-mutS1-luc	F: GCTGCTGGCCGGAAATCGAGGATTACCGG<br>R: CCGGTAATCCTCGATTTCCGGCCAGCAGC
Esrra-ΔDBD	F: CGCGGATCCAAGACAGCAGCCCCAGTGAAT<br>R: CCGGAATTCTCAGTCCATCATGGCCTCGAGC

Supplementary Table 2.   Primer sequences of ChIP-qPCR

Primer name	Sequence 5'- 3'
Leptin R1	F: TGGCCGGACCTCGAGGATTA
	R: CTTGCGCAACTGTCCGGC
Leptin R2	F: GCAGGTGCATTCTGTGATGTC
	R: GCTCTTTGCATACCTAACCTGG
Leptin R3	F: gTTTCCTCCCATTAggAACCCA
	R: CgAAGGTCGCAAGTGTGTTT
Spp1 R1	F: CCAACTGACCTGGAACACAGT
	R: GTGGCTCTGTTTTGTACTCCG
Spp1 R2	F: AGCAACAAGGTTCACGAGGT
	R: TATGCAGCCGCTTGCTCTTT

Supplementary Table 3. Primer sequences for qRT-PCR

Primer name	Sequence 5’-3’
Mouse *18s*	F: TAAGTCCCTGCCCTTTGTACACA
	R: GATCCGAGGGCCTCACTAAAC
Mouse *Esrra*	F: CTCAGCTCTCTACCCAAACGC
	R: CCGCTTGGTGATCTCACACTC
Mouse *Adipoq*	F: TGTTCCTCTTAATCCTGCCCA
	R: CCAACCTGCACAAGTTCCCTT
Mouse *Lep*	F: GAGACCCCTGTGTCGGTTC
	R: CTGCGTGTGTGAAATGTCATTG
Mouse *Spp1*	F: AGCAAGAAACTCTTCCAAGCAA
	R: GTGAGATTCGTCAGATTCATCCG
Mouse *Pparg*	F: TCGCTGATGCACTGCCTATG
	R: GAGAGGTCCACAGAGCTGATT
Mouse *Cebpa*	F: CCGTGGTGGTTTCTCCTTGA
	R: TCATTTTTCTCTCACGGGGCCA
Mouse *Fabp4*	F: TGAAATCACCGCAGACGACA
	R: ACACATTCCACCACCAGCTT
Mouse *Sp7*	F: ATGGCGTCCTCTCTGCTTG
	R: TGAAAGGTCAGCGTATGGCTT
Mouse *Bglap*	F: CCATCTTTCTGCTCACTCT
	R: GTCTGTTCACTACCTTATTGC
Mouse *Runx2*	F: AGAGTCAGATTACAGATCCCAGG
	R: TGGCTCTTCTTACTGAGAGAGG
Mouse *Vegfa*	F: GCACATAGAGAGAATGAGCTTCC
	R: CTCCGCTCTGAACAAGGCT
Human *SPP1*	F: GAAGTTTCGCAGACCTGACAT
	R: GTATGCACCATTCAACTCCTCG
Human *LEP*	F: TGCCTTCCAGAAACGTGATCC
	R: CTCTGTGGAGTAGCCTGAAG
