# Supplementary material for: Targeting adipocyte ESRRA promotes osteogenesis and vascular formation in adipocyte-rich bone marrow
Source: Nat Commun. 2024 May 4;15:3769. doi: 10.1038/s41467-024-48255-8 (PMC11069533; doi:10.1038/s41467-024-48255-8)
Supplement: Supplementary file 3 — Reporting Summary [file 41467_2024_48255_MOESM3_ESM.pdf]

nature portfolio

Corresponding author(s): Min Guan

Last updated by author(s): Mar 13, 2024

Reporting Summary

Nature Portfolio wishes to improve the reproducibility of the work that we publish. This form provides structure for consistency and transparency in reporting. For further information on Nature Portfolio policies, see our Editorial Policies and the Editorial Policy Checklist.

Please do not complete any field with "not applicable" or n/a. Refer to the help text for what text to use if an item is not relevant to your study. For final submission: please carefully check your responses for accuracy; you will not be able to make changes later.

Statistics

For all statistical analyses, confirm that the following items are present in the figure legend, table legend, main text, or Methods section.

n/a	Confirmed	
☐	☒	The exact sample size (n) for each experimental group/condition, given as a discrete number and unit of measurement
☐	☒	A statement on whether measurements were taken from distinct samples or whether the same sample was measured repeatedly
☐	☒	The statistical test(s) used AND whether they are one- or two-sided<br>*Only common tests should be described solely by name; describe more complex techniques in the Methods section.*
☒	☐	A description of all covariates tested
☐	☒	A description of any assumptions or corrections, such as tests of normality and adjustment for multiple comparisons
☐	☒	A full description of the statistical parameters including central tendency (e.g. means) or other basic estimates (e.g. regression coefficient) AND variation (e.g. standard deviation) or associated estimates of uncertainty (e.g. confidence intervals)
☐	☒	For null hypothesis testing, the test statistic (e.g. F, t, r) with confidence intervals, effect sizes, degrees of freedom and P value noted<br>*Give P values as exact values whenever suitable.*
☒	☐	For Bayesian analysis, information on the choice of priors and Markov chain Monte Carlo settings
☒	☐	For hierarchical and complex designs, identification of the appropriate level for tests and full reporting of outcomes
☒	☐	Estimates of effect sizes (e.g. Cohen's d, Pearson's r), indicating how they were calculated

Our web collection on statistics for biologists contains articles on many of the points above.

Software and code

Policy information about availability of computer code

Data collection	RNA-seq libraries were constructed and sequenced using an Illumina Novaseq™ 6000 by LC-Bio Technologies CO., LTD. (Hanzhou, China)<br>Micro-CT scanned was used Bruker Skyscan 1176.<br>RT-PCR data were collected using Analytik Jena qTOWER3.<br>Immunoblot results were collected using BioRad ChemiDoc XRS chemiluminescence imaging system.<br>Histological staining and immunofluorescence images were collected using fluorescent microscope (Olympus #BX53)
Data analysis	Genes differential expression analysis was performed by DESeq2 software.<br>Graph Pad Prism Version 8.0.1 and SPSS Statistics Version 26.0 were used for statistical analysis.<br>Micro-CT images were analyzed with NRecon version 1.6, CTvox version 2.2, and Dataviewer version 1.5.<br>Histological staining and immunofluorescence quantification were analyzed by using image J version 1.53k<br>Part of the schematic was produced in the website BioRender.com (with permission).

For manuscripts utilizing custom algorithms or software that are central to the research but not yet described in published literature, software must be made available to editors and reviewers. We strongly encourage code deposition in a community repository (e.g. GitHub). See the Nature Portfolio guidelines for submitting code & software for further information.

Data

Policy information about availability of data

All manuscripts must include a data availability statement. This statement should provide the following information, where applicable:

- Accession codes, unique identifiers, or web links for publicly available datasets
- A description of any restrictions on data availability
- For clinical datasets or third party data, please ensure that the statement adheres to our policy

The RNA sequencing generated in the present manuscript is available at https://www.ncbi.nlm.nih.gov/geo/query/acc.cgi?acc=GSE248799. The remaining data are available within the Article, and Supplementary information. Source data are provided with this paper.

Research involving human participants, their data, or biological material

Policy information about studies with human participants or human data. See also policy information about sex, gender (identity/presentation), and sexual orientation and race, ethnicity and racism.

Reporting on sex and gender	N/A
Reporting on race, ethnicity, or other socially relevant groupings	N/A
Population characteristics	N/A
Recruitment	N/A
Ethics oversight	N/A

Note that full information on the approval of the study protocol must also be provided in the manuscript.

Field-specific reporting

Please select the one below that is the best fit for your research. If you are not sure, read the appropriate sections before making your selection.

☒ Life sciences ☐ Behavioural & social sciences ☐ Ecological, evolutionary & environmental sciences

For a reference copy of the document with all sections, see nature.com/documents/nr-reporting-summary-flat.pdf

Life sciences study design

All studies must disclose on these points even when the disclosure is negative.

Sample size	No statistical methods were used to determine sample size. We used generally accepted sample sizes in accordance to own previous experiences with reproducable differences between conditions indicating that the chosen sample sizes were sufficient. Our sample sizes are similar to those generally employed in the life sciences field. We normally utilized 6-7 mice/group. In cell experiments, at least 3 biological independent samples in each group were used for detection in different experiments. The detailed sample size for each experiment is shown in all the figure legends.
Data exclusions	No data were excluded.
Replication	Once instruments and protocols were established, all attempts at replication were successfully reproduced. All data were replicated at least 3 times. Number of reproductions of each experimental finding is stated in figure legends.
Randomization	All samples were analyzed and allocated randomly. Mice with desired genotypes/treatments were randomly chosen for analysis
Blinding	Both in vitro cells and in vivo mice allocations were conducted in a blinded manner. Transwell migration and Matrigel tube formation assays, animal histological analysis, histomorphometric measurement, and immunofluorescence staining analysis were all done in a blinded manner.

Reporting for specific materials, systems and methods

We require information from authors about some types of materials, experimental systems and methods used in many studies. Here, indicate whether each material, system or method listed is relevant to your study. If you are not sure if a list item applies to your research, read the appropriate section before selecting a response.

Materials & experimental systems

n/a	Involved in the study
	☒ Antibodies
	☒ Eukaryotic cell lines
☒	Palaeontology and archaeology
	☒ Animals and other organisms
☒	Clinical data
☒	Dual use research of concern
☒	Plants

Methods

n/a	Involved in the study
☒	ChIP-seq
☒	Flow cytometry
☒	MRI-based neuroimaging

Antibodies

Antibodies used

1. Antibodies used in Western Blot:
anti-SPP1 (Novus Biologicals #AF808, 1:1000, diluted with 5% w/v BSA), anti-ESRRA (Cell Signaling Technology #13826, 1:1000, diluted with 5% w/v BSA), anti-Leptin (Abcam #ab16227,1:500; diluted with 5% w/v BSA), anti-GAPDH (Proteintech #60004-1-Ig , 1:5000, diluted with 5% w/v BSA), anti-Tubulin (BPI #AbM9005-37B-PU, 1:5000, diluted with 5% w/v BSA), HRP-goat anti-mouse IgG (EarthOx Life #E030110-02, 1:2000, diluted with 5% w/v BSA), HRP-goat anti-rabbit IgG (EarthOx Life #E030120-02, 1:2000, diluted with 5% w/v BSA) , HRP-rabbit anti-goat IgG (ABclonal #AS029; 1:1000, diluted with 5% w/v BSA).
2. Antibodies used in immunohistochemistry staining:
anti-PLIN1 (Cell Signaling Technology #9349, 1:200， diluted with 1% w/v BSA), anti-SPP1 (Novus Biologicals #AF808, 1:200， diluted with 1% w/v BSA), anti-Osterix (Abcam #ab209484, 1:200; diluted with 1% w/v BSA), anti-Endomucin (V.7C7) (EMCN, Santa Cruze #sc-65495,1:50; diluted with 1% w/v BSA), anti-CD31/PECAM-1 Alexa Fluor 488-conjugated Antibody (R&D system #FAB3628G, 1:200; diluted with 1% w/v BSA), anti-Leptin (Abcam #ab16227,1:200; diluted with 1% w/v BSA), Alexa Fluor 488-conjugated donkey anti-goat IgG (ThermoFisher #A-11055, 1:500; diluted with 1% w/v BSA), Alexa Fluor 555-conjugated donkey anti-rabbit IgG (1:500; Abcam #ab150074, diluted with 1% w/v BSA), Alexa Fluor 555-conjugated goat anti-rat IgG (Bioss #bs-0293G-AF555, 1:500; diluted with 1% w/v BSA).
3. Antibodies used in ChIP:
anti-ESR1 (Abcam #ab32063, 2 μl/IP), anti-ESRRA (Cell Signaling Technology #13826, 10 μl/IP), normal rabbit IgG (Cell Signaling Technology #2729, 1 μl/IP).

Validation

All antibodies in this study are commercially available and have been validated by manufacturer. Any validation statements are available on the manufacturer's website.
anti-SPP1 (Novus Biologicals #AF808) (https://www.novusbio.com/products/osteopontin-opn-antibody_af808)
anti-ESRRA (Cell Signaling Technology #13826) (https://www.cellsignal.cn/products/primary-antibodies/erra-e1g1j-rabbit-mab/13826)
anti-Leptin (Abcam #ab16227) (https://www.abcam.cn/products/primary-antibodies/leptin-antibody-ab16227.html)
anti-GAPDH (Proteintech #60004-1-Ig) (https://www.ptgcn.com/products/GAPDH-Antibody-60004-1-Ig.htm)
anti-Tubulin (BPI #AbM9005-37B-PU) (http://www.proteomics.org.cn/product/179.html)
anti-ESR1 (Abcam #ab32063) (https://www.abcam.cn/products/primary-antibodies/estrogen-receptor-alpha-antibody-e115-chip-grade-ab32063.html)
anti-PLIN1 (Cell Signaling Technology #9349) (https://www.cellsignal.cn/products/primary-antibodies/perilipin-1-d1d8-xp-rabbit-mab/9349)
anti-Osterix (Abcam #ab209484) (https://www.abcam.cn/products/primary-antibodies/sp7--osterix-antibody-epr21034-ab209484.html)
normal rabbit IgG (Cell Signaling Technology #2729) (https://www.cellsignal.cn/products/primary-antibodies/normal-rabbit-igg/2729)
anti-Endomucin (V.7C7) (EMCN, Santa Cruze #sc-65495) (https://www.scbt.com/zh/p/endomucin-antibody-v-7c7)
anti-CD31/PECAM-1 Alexa Fluor 488-conjugated Antibody (R&D system #FAB3628G) (https://www.rndsystems.com/cn/products/mouse-rat-cd31-pecam-1-alexa-fluor-488-conjugated-antibody_fab3628g)
HRP-goat anti-mouse IgG (EarthOx Life #E030110-02) (http://canlifesci.com/Product/Detail.aspx?Id=6161)
HRP-goat anti-rabbit IgG (EarthOx Life #E030120-02) (http://www.canlifesci.com/Product/Detail.aspx?Id=6162)
HRP-rabbit anti-goat IgG (ABclonal #AS029) (https://abclonal.com.cn/catalog/AS029)
Alexa Fluor 488-conjugated donkey anti-goat IgG (ThermoFisher #A-11055) (https://www.thermofisher.cn/cn/zh/antibody/product/Donkey-anti-Goat-IgG-H-L-Cross-Adsorbed-Secondary-Antibody-Polyclonal/A-11055)
Alexa Fluor 555-conjugated donkey anti-rabbit IgG (Abcam #ab150074) (https://www.abcam.cn/products/secondary-antibodies/donkey-rabbit-igg-hl-alexa-fluor-555-ab150074.html)
Alexa Fluor 555-conjugated goat anti-rat IgG (Bioss #bs-0293G-AF555) (http://www.bioss.com.cn/prolook_03.asp?id=AF08169606011873&pro37=4)

Eukaryotic cell lines

Policy information about cell lines and Sex and Gender in Research

Cell line source(s)

Primary BMSCs were isolated from male mouse femur. 3T3-L1 (ATCC® CL-173™). Human BMSCs (Cyagen Biosciences Inc. (#HUXMF-01001)).

Authentication

Each cell line used was morphologically confirmed according to the information provided by culture collections. Human BMSCs were observed to have a high level of purity, with > 70% expressing CD29, CD44, and CD105, and <5% expressing CD34 and CD45 in flow cytometry assays, implemented by Cyagen Biosciences Inc.

Mycoplasma contamination	Cells were routinely tested and found negative for mycoplasma contamination.
Commonly misidentified lines (See ICLAC register)	None

Animals and other research organisms

Policy information about studies involving animals; ARRIVE guidelines recommended for reporting animal research, and Sex and Gender in Research

Laboratory animals	Homozygous Esrraflox/flox mice with loxP sites in the same orientation at both ends of exon 2 of the Esrra gene were previously generated by Shanghai Model Biology Center Inc. The Adipoq-Cre mice [B6; FVB-Tg(Adipoq-cre)1Evdr/J] (JAX Strain: 010803) were obtained from the National Resource Center for Mutant Mice of China. The Cre/loxP system was used to generate adipocyte-specific Esrra knockout mice (Adipoq-Cre; Esrrafl/fl, referred to as EsrraAKO) by consecutive mating of Esrraflox/flox mice with AdipoqCre mice. The Cre-negative Esrraflox/flox (Esrrafl/fl) mice were employed as the control genotype.<br>9-week-old male Esrrafl/fl and EsrraAKO mice were fed either a normal chow diet (NCD) or a high-fat diet (HFD, 60% kcal in fat (about 35% fat); D12492, Research Diets) for up to 16 weeks. All of the mice were euthanized at the age of 25 weeks.<br>10-week-old female Esrrafl/fl and EsrraAKO mice were subjected to either bilateral ovariectomy to mimic postmenopausal osteoporosis or sham operation. The mice were euthanized at the age of 18 weeks.<br>6-week-old male C57BL/6 mice were purchased from GemPharmatech (Nanjing, China). 7-week-old male mice were randomized into two groups: NCD and DIO groups. The DIO mice were fed with a high-fat diet for 14 weeks to induce obesity, and they were then divided into two subgroups: the DIO+C29 group and the DIO+Veh group. The mice in DIO+C29 group were administered an ESRRA inverse agonist C29 via oral gavage at a dose of 30 mg/kg body weight daily for 4 weeks. The DIO+Veh group was administered an equal volume of vehicle. Both DIO+Veh group and DIO+C29 group were exposed to 4 additional weeks of HFD feeding and treatments. Mice in NCD+Veh group were fed a regular chow diet throughout the experiment and were administered the equal volume of vehicle as described in DIO+Veh group by oral gavage for the last 4 weeks. The mice were euthanized at the age of 25 weeks.<br>All mice were maintained in a specific-pathogen-free facility at 24± 2°C with 60% ± 5% humidity under 12 h light/dark cycles which was defined as normal conditions.
Wild animals	No wild animals were used in the study.
Reporting on sex	For the NCD and DIO experiments, male Esrrafl/fl and EsrraAKO mice were used.<br>For Compound29 treatment experiments, male C57BL/6 were used.<br>For the sham and OVX experiments, female Esrrafl/fl and EsrraAKO mice were used.
Field-collected samples	No field collected samples were used in this study.
Ethics oversight	All procedures used for animals and their care in this study were reviewed and approved by the ethical committee at Shenzhen Institute of Advanced Technology, Chinese Academy of Sciences.

Note that full information on the approval of the study protocol must also be provided in the manuscript.

Plants

Seed stocks	N/A
Novel plant genotypes	N/A
Authentication	N/A

This checklist template is licensed under a Creative Commons Attribution 4.0 International License, which permits use, sharing, adaptation, distribution and reproduction in any medium or format, as long as you give appropriate credit to the original author(s) and the source, provide a link to the Creative Commons license, and indicate if changes were made. The images or other third party material in this article are included in the article's Creative Commons license, unless indicated otherwise in a credit line to the material. If material is not included in the article's Creative Commons license and your intended use is not permitted by statutory regulation or exceeds the permitted use, you will need to obtain permission directly from the copyright holder. To view a copy of this license, visit http://creativecommons.org/licenses/by/4.0/
